# Influence of Osmotic Pressure on Nanostructures in Thin Films of a Weakly-Segregated Block Copolymer and Its Blends with a Homopolymer

**DOI:** 10.3390/polym13152480

**Published:** 2021-07-28

**Authors:** Yi-Fang Chen, Jia-Wen Hong, Jung-Hong Chang, Belda Amelia Junisu, Ya-Sen Sun

**Affiliations:** Department of Chemical and Materials Engineering, National Central University, Taoyuan 32001, Taiwan; flower840227@gmail.com (Y.-F.C.); bessieee@g.ncu.edu.tw (J.-W.H.); jasograce0123@gmail.com (J.-H.C.); beldaamelia@g.ncu.edu.tw (B.A.J.)

**Keywords:** surface wrinkling, thin film, block copolymer

## Abstract

We studied the influence of osmotic pressure on nanostructures in thin films of a symmetric weakly-segregated polystyrene-block-poly (methyl methacrylate), P(S-b-MMA), block copolymer and its mixtures with a polystyrene (PS) homopolymer of various compositions. Thin films were deposited on substrates through surface neutralization. The surface neutralization results from the PS mats, which were oxidized and cross-linked by UV-light exposure. Thus, thermal annealing produced perpendicularly oriented lamellae and perforated layers, depending on the content of added PS chains. Nevertheless, a mixed orientation was obtained from cylinders in thin films, where a high content of PS was blended with the P(S-b-MMA). A combination of UV-light exposure and acetic acid rinsing was used to remove the PMMA block. Interestingly, the treatment of PMMA removal inevitably produced osmotic pressure and consequently resulted in surface wrinkling of perpendicular lamellae. As a result, a hierarchical structure with two periodicities was obtained for wrinkled films with perpendicular lamellae. The formation of surface wrinkling is due to the interplay between UV-light exposure and acetic acid rinsing. UV-light exposure resulted in different mechanical properties between the skin and the inner region of a film. Acetic acid rinsing produced osmotic pressure. It was found that surface wrinkling could be suppressed by reducing film thickness, increasing PS content and using high-molecular-weight P(S-b-MMA) BCPs.

## 1. Introduction

The self-assembly of diblock copolymers (BCPs) in bulk has been well established [1]. The equilibrium phase behavior in bulk is governed by segregation strength (χN) and volume fractions (*f*) of constituent blocks, where χ is the Flory-Huggins interaction and N is the degree of polymerization. Below an order-disorder transition temperature, the self-assembly of BCPs can yield a variety of nanodomains of one-, two-, and three-dimensional periodicities [1]. However, as BCPs are confined to thin films, surface fields (at air/BCP and substrate/BCP interfaces) and geometric confinement factors play important roles in the phase behavior, which deviates from the phase behavior in bulk [2]. The two factors have been thoroughly studied for cylinder-forming [3,4,5,6,7,8] or lamellae-forming [9,10,11,12] BCP systems. For example, if an asymmetric BCP that forms cylinders in bulk is confined to thin films with symmetric or asymmetric wetting interfaces, tuning the surface fields and/or changing the film thickness can form several non-cylinder morphologies, such as wetting lamellae, perforated layers, spheres and even the coexistence of multiple phases. The non-cylinder structures can laterally coexist at the same depth or vertically coexist at different depths [3,4,5,6,7]. In addition to morphological diversity, domain orientation, shape or size undulation are also influenced by both spatial confinement and surface fields [2,3,4,5,6,7,8,9,10,11,12,13,14,15,16,17,18,19,20,21].

Adding a homopolymer into a BCP melt not only causes morphological changes but also affects the relative stability of various morphologies [22,23,24,25,26,27,28,29]. Morphological changes are a result of variations in the volume fraction of the constituent block. New phases can stabilize by homopolymer chains filling the space originally occupied by highly stretched copolymer chains [25,26]. Furthermore, adding a homopolymer into BCPs can increase morphological diversity, amplify size tunability and enhance phase stability [22,23,24,25,26,27,28,29]. Hence, an introduction of spatial confinement and surface fields into BCP/homopolymer mixtures can even lead to an increased level of complexity for self-assembly in thin films [30,31,32,33,34,35,36,37,38,39]. Furthermore, the addition of a homopolymer also influences the kinetic and dynamic spatial order of nanodomains in thin films [40,41].

Surface wrinkling has recently generated considerable research interest because it results in a series of surface morphologies on the micrometer scale. Surface wrinkling has been studied for homogenous films deposited on a soft substrate, homogenous gels with solvent swelling, and bilayer films with two distinct mechanical properties [42,43]. Nevertheless, little attention has been paid to BCP thin films [44,45,46]. One of the driving forces which induces surface wrinkling arises from osmotic pressure. Osmotic pressure can partly exist due to solvent swelling when polymer films are immersed in an organic solvent or acid aqueous solution. Thus, a combination of osmotic pressure and BCP self-assembly has been used to produce hierarchical structures [44,45,46,47] or porous membranes [39,48,49], for which BCP itself cannot solely form through self-assembly.

Surface wrinkling has been found in thin films of other BCPs [44,45,46]. However, as far as we know, surface wrinkling has not yet been reported for the P(S-*b*-MMA) system. A combination of UV-light exposure and acetic acid rinsing [39,48], or a single step of acetic acid rinsing [50,51], has been frequently used to make porous membranes from P(S-*b*-MMA) films. In some cases, film instability may occur during such processes. This study aims to understand the effects of osmotic pressure on nanostructures in thin films on substrates with surface neutralization. The surface neutralization results from the PS mats, which were oxidized and cross-linked by UV-light exposure. The thin films were composed of a P(S-b-MMA) and its miscible mixtures with a homopolymer polystyrene (PS) of various weight fractions. The homopolymer PS used for preparing P(S-b-MMA)/PS mixtures has a lower molecular weight than the PS and PMMA blocks. Upon finely tuning the blending compositions, several morphologies with perpendicular or mixed orientation were obtained in thin film self-assembly for the P(S-b-MMA)/PS mixtures, on substrates with neutral surface. A combination of UV-light exposure and acetic acidic rinsing was applied to remove the PMMA component. Interestingly, solvent swelling inevitably resulted in osmotic pressure. The osmotic pressure could cause surface wrinkles in perpendicular lamellae. As a result of osmotic pressure, a hierarchical structure with two periodicities formed in wrinkled films containing lamellae. Nevertheless, such osmotic pressure only produced dimple-like or mushroom-like pores in the films that formed perforated layers and cylinders. Mechanisms of surface wrinkling of lamellae are detailed in the last section.

## 2. Experimental Section

### 2.1. Materials and Sample Preparation

Five symmetric polystyrene-*block*-poly(methyl methacrylate) BCPs, P(S-b-MMA), and two polystyrene (PS) homopolymers were purchased from Polymer Source Inc. and used as received. The five block copolymers are briefly denoted as P(S_x_-*b*-M_y_), where x and y denote values of molecular weight in unit of kg/mol, if specified. Details of the five BCPs and two homopolymers are given in Table 1. The high-molecular-weight, PS_270_, was used for surface neutralization. The low-molecular-weight, PS_6_, was used to prepare P(S_21_-b-M_21_)/PS_6_ mixtures. P(S_21_-b-M_21_) was first dissolved in toluene to prepare BCP solutions. Various amounts of PS_6_ were then added into the solutions to give P(S_21_-b-M_21_)/PS_6_ mixtures in toluene. Total polymer concentrations in toluene were 1.5 and 3 wt%, respectively. Three weight fraction ratios were prepared for the P(S_21_-b-M_21_)/PS_6_ mixtures; P(S_21_-b-M_21_)/PS_6_ = 95/5, 75/25, and 50/50. The volumetric fractions (ϕ_PS_) of the PS constituent in the mixtures correspond to ϕ_PS_ = 55, 64, and 77 vol%, respectively. Films of neat P(S-*b*-MMA) and P(S_21_-b-M_21_)/PS mixtures were prepared by spin-coating the solutions onto substrates. The thickness of the films was controlled by using different polymer concentrations and spin rates. Two thicknesses (*h*_i_) initially obtained were approximately 16.3 nm (1.5 wt%, 5000 rpm) and 97 nm (3 wt%, 3000 rpm), respectively. After one-day dying, all the as-spun films of binary P(S-*b*-MMA)/PS_6_ mixtures were annealed at 230 °C for 1 h.

To prepare surface-neutralized SiO_x_/Si substrates, we used PS_270_ as a capping layer for surface neutralization. Each capping layer on SiO_x_/Si was prepared by spin coating (4000 rpm, 1 min) from a solution containing 1 wt% PS_270_ in toluene. After complete removal of the toluene, the PS_270_-covered SiO_x_/Si substrates were exposed to UV-light exposure (at λ = 254 nm) in a glove box for cross-linking and mild oxidation of the PS_270_ capping layer. The thickness of the PS_270_ capping layer was approximately 24 nm. Details of the treatment of UV-light exposure, used for the neutralization of the substrate interface, have been reported elsewhere [52,53,54]. After cross-linking and mild oxidation, the PS_270_ homogeneous layers were resistant to solvents. The contact angle of a water droplet exhibited 78~82° on the surface-neutralized SiO_x_/Si substrates. For the contact-angle range, there should be no affinity between the substrates and the constituent blocks. For brevity, PS_270_^n^ denotes the neutralized PS_270_ layers.

To increase the contrast between PS and PMMA blocks in thin films, the PMMA component was selectively degraded, and the PS component was cross-linked by UV-light exposure in a glove box, filled with Ar or N_2_ gas for 2.5 h (λ = 254 nm; UV-light dosage = 2.7 mW/cm^2^). The degraded PMMA component was then quickly removed by short immersion (10 s) in acetic acid.

### 2.2. Materials Characterization

For the thin films of neat P(S_21_-b-M_21_) and its mixtures with various contents of PS, surface morphologies were characterized by a three real-space technique: an optical microscope (OM, Olympus BX-BLA2) in reflection mode, an atomic force microscope (AFM, SPA400 Seiko, Tokyo, Japan) in tapping mode, and a field-emission scanning electron microscope (FE-SEM, SUB8200 Hitachi, Tokyo, Japan) performed at 10 keV. The thermally annealed films, which were deposited on the PS_270_^n^-capped SiO_x_/Si substrate, were characterized by grazing-incidence small-angle X-ray scattering (GISAXS) at BL 23A of the National Synchrotron Radiation Research Center (NSRRC), Hsinchu, or by in-house GISAXS instrument (Nano-Viewer, Rigaku, Tokyo, Japan), using Cu K_α_ X-rays of λ = 1.54 Å (30 kV and 40 mA) at the National Central University (NCU). The incident angle was 0.17° and the energy of synchrotron-based X-rays was 10 keV for GISAXS measurements at NSRRC. Reciprocal-space scattering signals were collected by a Pilatus 2D area detector. The output data are GISAXS 2D images with intensity as functions of q_//_ and q_⊥_, where q_//_ and q_⊥_ denote the scattering momentum vector along the lateral and normal direction, respectively. A standard sample, silver behenate, was used for calibration of the sample-to-detector distance and scattering momentum vector.

## 3. Results and Discussion

### 3.1. Morphological Observations

Our previous studies [52,53,54] have demonstrated that cylinders and lamellae tend to adopt perpendicular orientations to SiO_x_/Si with a layer of neutralized PS chains. For the films on PS_270_^n^-SiO_x_/Si, there is no miscibility between the PS_270_^n^ and the PS block because the molecular weight of PS_270_^n^ is considerably higher than the molecular weight of the PS block. In parallel to Hashimoto et al.’s findings [22,23], large disparities in chain length can lead to immiscibility rather than miscibility. Furthermore, after the capping layer of the PS_270_ chains was cross-linked and mildly oxidized, its chain mobility was limited and it was therefore not miscible with the short PS_6_ chains. Thus, after thermal annealing, there must be no interpenetration between the BCP films and the neutralized PS layers of the specimens.

**Figure 1:**
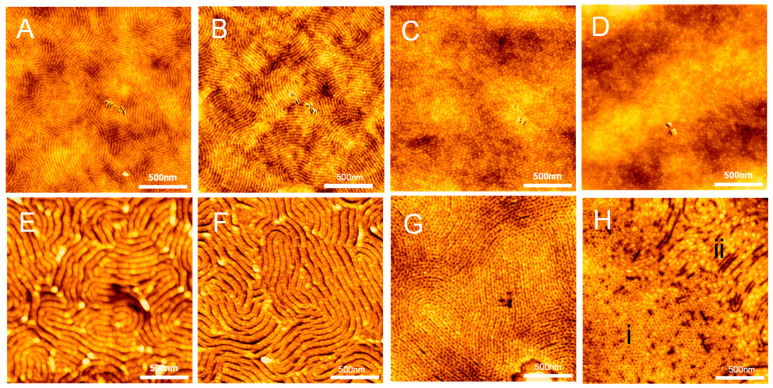
AFM topographic images of thin films (h_i_ = 97 nm) of P(S_21_-*b*-M_21_)/PS_6_ mixtures of various weight fractions: (**A**,**E**) 100/0, (**B**,**F**) 95/5, (**C**,**G**) 75/25 and (**D**,**H**) 50/50, respectively. The thin films were spin-coated on PS_270_^n^-capped SiO_x_/Si substrates and then annealed at 230 °C (1 h). Images A–D were collected for the films without a combination of UV-light exposure (2.5 h) and acetic acid rinsing. Images E–H were collected for the films through a combination of UV-light exposure (2.5 h) and acetic acid rinsing.

Figure 1A–D shows representative AFM images of the pristine thin films (h_i_ = 97 nm) without UV-light exposure and acetic acid rinsing. As Figure 1A–D shows, several prominent features are worth our attention. Firstly, the neat P(S_21_-b-M_21_) and P(S_21_-b-M_21_)/PS_6_ (95/5) thin films both display a morphology of fingerprint-like nanodomains on PS_270_^n^-SiO_x_/Si (Figure 1A,B). The P(S_21_-b-M_21_)/PS_6_ (75/25) film displays a morphology of dot-like nanodomains packed in rows (Figure 1C). In particular, each row of dot-like nanodomains is sandwiched by two neighboring worm-like nanodomains. This morphology appears to be similar to a morphology of discrete perforations within lamellae [55]. Furthermore, the dot-like nanodomains lack in-plane hexagonal arrays. For the P(S_21_-b-M_21_)/PS_6_ (50/50) film, the AFM image shows a patchy surface (Figure 1D). The patchy surface is partially due to the coexistence of dot-like nanodomains with an ill-defined morphology. The ill-defined morphology suggests that there should be a PS overlayer on the free surface. The PS overlayer has an incomplete surface coverage. Thus, the patchy PS overlayer may be due to partial surface aggregation from short PS_6_ chains. Such surface aggregation of short chains in our system is similar to the segregation behavior of short chains in a binary mixture of two homopolymers with different molecular weights [56,57].

Nevertheless, with the exception of the P(S_21_-b-M_21_)/PS_6_ (50/50) film, there is no PS overlayer on the surface of most of the thin films on PS_270_^n^-SiO_x_/Si. Thus, the PMMA nanodomains are directly exposed to air. To increase the morphological contrast of the thin films on PS_270_^n^-SiO_x_/Si, UV-light exposure of 2.5 h was performed in order to selectively degrade the PMMA block and to cross-link the PS block. After UV-light exposure, the degraded PMMA block chains were rinsed by brief immersion in acetic acid. Upon removal of degraded PMMA through acetic acid rinsing, a hierarchical structure was obtained from the structural evolution of the fingerprint-like nanodomains (Figure 1E,F). Furthermore, acetic acid rinsing not only removed the PMMA nanodomains but also “swelled” the PS nanodomains. Close inspection of Figure 1E-F demonstrates that the hierarchical structure was composed of two periodic series of alternating layers. The first periodic series was alternating layers of relief nano-stripe and shallow nanogroove; the second periodic series was composed of alternating layers of deep nanogroove and relief mesa.

We further analyzed the fast Fourier transfer (FFT) patterns and height profiles of AFM images 1A&B and 1E&F (Figures S1 and S2 of supporting information). As one can see, the hierarchical structure exhibits two periodic spacings (Figure S1C,D) but the fingerprint-like nanodomains only show one periodic spacing (Figure S1A,B). A careful comparison indicates that the small spacing should result from the intrinsic periodicity of the self-assembled nanodomains, while the large spacing should result from the correlation of the deep nanogrooves.

Figure S2 shows the height profiles of the neat P(S_21_-b-M_21_) and P(S_21_-b-M_21_)/PS_6_ (95/5) thin films before and after a combination of UV-light exposure and acetic acid rinsing. As Figure S2A,B shows, the height difference between the relief nano-stripes and shallow nanogrooves was less than 2 nm before UV-light exposure followed by acetic acid ringing. In contrast, the height difference between the relief mesas and deep trenches became more than 8 nm after UV-light exposure followed by acetic acid ringing (Figure S2C,D). Furthermore, the two periodic spacings were incommensurate. In other words, d_2_ ≠ nd_1_, where d_1_ denotes the spacing of the self-assembled nanodomains, d_2_ denotes the spacing of the deep nanogrooves, and n is an integer. d_1_ and d_2_ were approximately evaluated as 26.1–29.9 nm and 78.3–84.1 nm, respectively.

**Figure 2:**
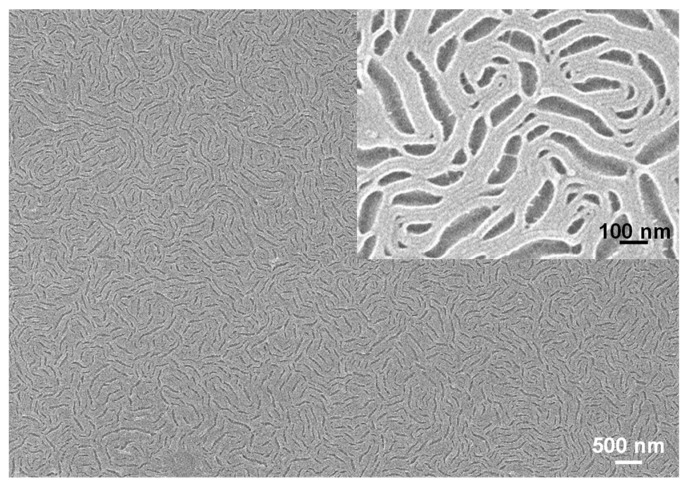
Low- and high-magnification SEM images of a P(S_21_-b-M_21_) film of h_i_ = 97 nm. The film was prepared under the same procedure described in the previous figure caption.

To clearly see the pattern of a hierarchical structure in the two periodicities, we characterized another neat PS-b-PMMA film of hi = 97 nm with SEM. The film was prepared by the same preparation procedure as the sample in Figure 1E. As the low-magnification SEM image (Figure 2) shows, the surface of the film clearly displays a similar morphology of hierarchical structures as the two periodicities. Close scrutiny of the SEM image shows that the lower periodicity is produced by the long period of perpendicular lamellae (see the insert of Figure 2).

The P(S_21_-b-M_21_)/PS_6_ (75/25) thin film on PS_270_^n^-SiO_x_/Si displays another striking morphology, after the same procedure was carried out to remove PMMA but crosslink PS (Figure 1G). As Figure 1G shows, alternating layers and rows of dimple-like pores coexisted. Each row of dimple-like pores was surrounded by two neighboring layers. In contrast, the same procedure produced a coexistence of two kinds of nanostructures for the P(S_21_-b-M_21_)/PS_6_ (50/50) film on PS_270_^n^-SiO_x_/Si. As Figure 1H shows, one kind composed dimple-like pores (marked as region i) and the other kind composed mushroom-like nanodomains (marked as region ii). The dimple-like pores were present in the areas that initially displayed dot-like nanodomains, while the mushroom-like nanodomains were present in the patchy areas that displayed the ill-defined morphology. Close inspection of the morphology of region ii demonstrates that the mushroom-like nanodomains seem to grow on top of worm-like nanodomains.

The observed morphologies are further explained in detail. Firstly, three phases formed at different PS contents. Similarly to the neat symmetric P(S_21_-b-M_21_), the P(S_21_-b-M_21_)/PS_6_ (95/5) thin films formed lamellae. The P(S_21_-b-M_21_)/PS_6_ (75/25) films formed perforated layers. The P(S_21_-b-M_21_)/PS_6_ (50/50) films formed cylinders. Secondly, perpendicular domain orientation was favored for the lamellae and perforated-layer phases, but a mixed orientation was favored for the cylinder phase on PS_270_^n^-capped SiO_x_/Si. Thirdly, the hierarchical nanostructure obtained for the films with perpendicular lamellae may be linked to surface instability as a result of osmotic pressure. Furthermore, osmotic pressure also led to the formation of mushroom-like structures for the cylinders on PS_270_^n^-SiO_x_/Si. More details of surface wrinkling will be interpreted in the last section.

For the P(S_21_-b-M_21_)/PS_6_ (75/25) mixture, its thin films tend to form perforated layers where the PS component not only formed layers but also formed perforations embedded within the PMMA layers. During the perforated-layer phase, PMMA removal produced dimple-like nanodomains, as observed in Figure 1G. Close inspection of Figure 1G demonstrates that the perpendicular perforated layers on PS_270_^n^-capped SiO_x_/Si were dense in dislocation and disclination defects. The defects span over a large area along the lateral direction. Due to the defects, the PS perforations in the perpendicular perforated layers lacked a long-range order, and consequently neither AB nor ABC inter-layer stacking was present in the perforations. Instead, a liquid-like order appeared in the PS perforations.

The coexistence of patches of dimple-like pores and mushroom-like nanostructures suggests that the cylinders on PS_270_^n^-SiO_x_/Si should have two different orientations (Figure 1H). The coexistence of the mixed orientation is ascribed to the surface segregation of certain short PS chains. In other words, the free surface should not reach a perfect neutralization state when the P(S_21_-b-M_21_)/PS_6_ (50/50) film on PS_270_^n^-SiO_x_/Si is annealed at 230 °C. For thin films of neat P(S-*b*-MMA) BCPs, the neutralization of the free surface can be controlled by annealing the temperature [58].

As reported in literature [59], the surface energy (γ) of polymers is usually a function of temperature (*T*) and molecular weight (MW). γ is inversely proportional to *T* but directly proportional to MW. For a symmetric P(S-b-MMA) BCP, PS has a lower γ than PMMA at room temperature. The difference in γ is due to the intrinsic nature between the two chemically different species. As the temperature-dependent declining of γ is slightly higher for PMMA than for PS upon heating, the γ values of PS and PMMA can eventually become identical when thermal annealing is performed a high temperature [58,60].

For a symmetric, lamellae-forming P(S-b-MMA), the free surface can be perfectly neutralized by thermal annealing at 225 °C [58]. As both the free surface and substrate interfaces are perfectly neutralized, there must be no segregation of either of the two blocks onto the free surface and substrate interface. However, if there is any disparity in the molecular weight between the PS and PMMA blocks or between their chemically identical species, achieving a neutralized free surface with temperature control should compensate the additional disparity. Furthermore, Demarquette et al. demonstrated that the surface energy of PS decreases with increasing MW [59]. In our study, we speculate that the low-molecular-weight PS_6_ must have a lower γ than the PS block. Thus, selectively adding the low-molecular-weight PS_6_ homopolymer into the PS block should increase the disparity in the surface energy between the PMMA and PS blocks. Hence, annealing at 230 °C may not produce a perfect neutral situation for the free surface. In other words, the free surface should still remain selective for the affinity of the PS_6_. The competition between the selective free surface and the neutral substrate interface consequently led to the coexistence of the two orientations observed for the P(S_21_-b-M_21_)/PS_6_ (50/50) film on PS_270_^n^-SiO_x_/Si.

An alternative reason is that grains have different orientations in an ill-defined cylindrical structure, which might also produce the morphology observed in Figure 1D. This might be due to a poor neutralization (irradiation of the PS mats affords surface neutralization for lamellar morphology) or non-optimized annealing conditions (annealing conducted too close to the ODT due to the addition of the short PS chains). Considering that the above reason is also possible when interpreting the ill-defined morphology, the surface preference of short chains on thin blend films will be further clarified in the future.

The parallel PMMA cylinders should be buried underneath the free surface because of the surface segregation of certain short PS chains. In the presence of aggregated PS chains, the parallel PMMA cylinders underneath the free surface should still remain in a pristine state. This is because UV-light exposure was not able to degrade the PMMA block through the PS overlayer. Furthermore, UV-light exposure cross-linked the PS overlayer. Under this condition, immersion in acetic acid should produce an accumulation of osmotic pressure within the pristine PMMA cylinders. Once the magnitude of osmotic pressure overwhelms the mechanical property of the PS block, the PMMA cylinders swollen by acetic acid are forced to pierce the PS overlay, consequently resulting in mushroom cups. Such behavior is similar to the response of the nanostructures immersed in an acidic solution [47]. In contrast, the perpendicular PMMA cylinders on PS_270_^n^-SiO_x_/Si were able to be directly removed by the same combination of UV-light exposure and acetic acid rinsing. As a result, the removal of the perpendicular PMMA cylinders produced dimple-like nanodomains.

### 3.2. Spatial Ordering

**Figure 3:**
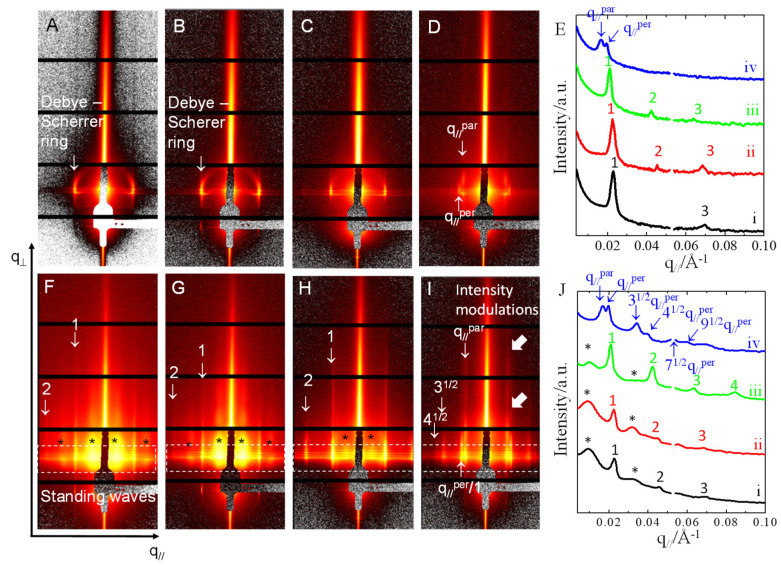
(**A**–**D**,**F**–**I**) GISAXS 2D patterns and (**E**,**J**) 1D profiles of thin films (*h*_i_ = 97 nm) of P(S_21_-b-M_21_)/PS_6_ mixtures of various weight fractions: (**A**,**E**_i_,**F**,**J**_i_) 100/0, (**B**,**E**_ii_,**G**,**J**_ii_) 95/5, (**C**,**E**_iii_,**H**,**J**_iii_) 75/25 and (**D**,**E**_iv_,**I**,**J**_iv_) 50/50, respectively. The films were spin-coated on PS_270_^n^-SiO_x_/Si and annealed at 230 °C for 1 h. Broad rods, corresponding to the scattering of wrinkles, are marked by black asteroids (*). The positions of the diffraction truncation rods are labelled by q_//_ ratios, and standing waves are highlighted by dotted boxes for a visual guide. In (**E**,**J**), lines i−iv correspond to the 1D GISAXS profiles extracted from in-plane scan cuts along the intense streaks. Broad humps, a scattering feature of wrinkles, are marked by black asteroids (*).

Figure 3 shows GISAXS 2D patterns of another set of P(S_21_-b-M_21_)/PS_6_ thin films with the same compositions as those that were prepared on PS_270_^n^-SiO_x_/Si for morphological observations. Figure 3 shows GISAXS 2D patterns and 1D profiles for the pristine films with no UV-light exposure or acetic acid rinsing. As Figure 3A,B shows, both the perpendicular lamellae and perforated layers supported on PS_270_^n^-SiOx/Si show partial Debye-Scherrer (DS) arcs in the 2D GISAXS patterns. Such DS arcs have been interpreted in previous studies [61,62,63]. The presence of DS arcs can be ascribed to the formation of defective lamellae. Thus, it is likely that some of the lamellae might not be perfectly perpendicular to the substrate direction, and that defective lamellae, such as tilted and/or kinked lamellae, may exist in the perpendicular lamellae. Quantitatively analyzing the 1D in-plane GISAXS profiles demonstrates that the perpendicular lamellae on PS_270_^n^-SiO_x_/Si exhibit diffraction peaks in series with a positional ratio of either 1:3 for neat P(S_21_-b-M_21_) or 1:2:3 for P(S_21_-b-M_21_)/PS_6_ (95/5) (Figure 3E_i_,E_ii_). The systematic forbidden of even-number diffraction peaks, as shown in Figure 3E_i_, is due to the symmetric volume fraction of the neat P(S_21_-b-M_21_). Forbidden of even-number diffraction peaks is common when two phases forming lamellae have equal volume fractions [64]. The emerging of even-number diffraction peaks, as shown in Figure 3E_ii_, is ascribed to compositional asymmetry resulted from a selective incorporation of a small amount of PS chains into the PS block. According to the position of the first-order peak, the long period of lamellae was estimated as 27.7 nm for the neat P(S_21_-b-M_21_) film and 27.9 nm for the P(S_21_-b-M_21_)/PS_6_ (95/5) film.

For the perpendicular perforated layers on PS_270_^n^-SiO_x_/Si, the 1D GISAXS profile only displays a feature of lamellar stacking, showing diffraction peaks in series with a positional ratio of 1:2:3 (Figure 3E_iii_). The result suggests that the perforations in the perpendicular perforated layers only have a liquid-like order so that only the diffraction feature corresponding to lamellar stacking is present. By quantitatively analyzing the first-order peak centered at q_//_ = 0.0211 Å^−1^, the inter-lamellae distance was estimated to be 29.8 nm for the perforated-layer phase.

For the P(S_21_-b-M_21_)/PS_6_ (50/50) film on PS_270_^n^-SiO_x_/Si, two sharp scattering rods are discerned (Figure 3D,E_iv_). The two rods have a positional ratio of 1:(4/3)^1/2^. According to Kramer et al.’s study [65], the positional ratio can be taken as an indication of cylinders with mixed perpendicular and parallel orientation. The two scattering rods are marked as q_//_^par^ and q_//_^per^, where q_//_^par^ denotes the first-order diffraction of parallel cylinders and q_//_^per^ denotes the first-order diffraction of perpendicular cylinders. The q_//_^par^ rod centered at 0.0170 Å^−1^ exhibits intensity undulations in series along the q_⊥_ direction but such intensity undulations are absent from the q_//_^per^ rod centered at 0.0197 Å^−1^ (Figure 3D,E_iv_).

After being exposed to UV-light irradiation and subsequent acetic acid rinsing, the films deposited on PS_270_^n^-SiO_x_/Si were characterized by GISAXS. As Figure 3F,J shows, several noticeable features emerge. Firstly, standing waves are clearly observable for the films. Secondly, the partial Debye-Scherrer rings disappeared for the films with perpendicular lamellae (Figure 3F,G). The absence of the Debye-Scherrer arcs implies that the defective lamellae might be eliminated by osmotic pressure. Thirdly, the sharp Bragg diffraction truncation rods extend toward high q_⊥_ with enhanced intensity. The enhanced intensity observed for those sharp rods is due to an increase in the scattering contrast between the porous channels and cross-linked PS nanodomains. Furthermore, there are additional diffraction lobes, which newly emerge at q_//_ = 0.0095 Å^−1^ and symmetrically locate beside the shadow of beam stopper. The diffraction lobes are highlighted by black asteroids in Figure 3F,G,J_i_,J_ii_. The additional diffraction lobes exhibit strong intensities and broad q_//_ widths. The broad diffraction lobes should arise from the wrinkles as a result of osmotic pressure that may form during acetic acid rinsing. The broad q_//_ width indicates that the wrinkles have a broad distribution of inter-domain spacing. The averaged inter-domain distance (λ_wrinkle_) was approximately 66.4 nm.

For the P(S_21_-b-M_21_)/PS_6_ (75/25) thin film, the Bragg diffraction rods display enhanced intensity so that the positional ratio of 1:2:3 can be easily recognized in the GISAXS pattern (Figure 3H,J_iii_). In addition, the broad hump associated with surface wrinkles shows a depressed intensity. The depressed intensity indicates that wrinkle formation could be suppressed in the perforated layers. The reason is that the incorporation of the PS homopolymer could increase inter-layer connectivity and may increase resistance to the deformation that was driven by osmotic pressure (Figure 3H,J_iii_).

Figure 3I shows GISAXS pattern of the P(S_21_-b-M_21_)/PS_6_ (50/50) thin film. The diffraction feature associated with wrinkles was totally absent, indicating that film wrinkling could be completely prohibited for the P(S_21_-b-M_21_)/PS_6_ (50/50) thin film. The absence of surface wrinkling is due to an increase in continuity and interconnectivity of the PS matrix. As a result, only intense diffraction truncation rods/peaks, corresponding to self-assembled nanocylinders, are left in Figure 3I,J_iv_. Even high-order peaks labelled by 3^1/2^, 4^1/2^, 7^1/2^ and 9^1/2^ emerged at high q_//_. The relative q_//_ positions offer evidence that the diffraction rods/peaks marked with q_//_^par^ in series resulted from parallel cylinders and the rods/peaks marked with q_//_^per^ resulted from perpendicular cylinders. Another striking feature shown in Figure 3I demonstrates that the intensity extension of the q_//_^par^ peak displays several modulations along the q_⊥_ direction. The intensity of modulations imply a heterogeneous density distribution along the substrate normal. In contrast, the q_//_^per^ rods exhibit no intensity modulations along the q_⊥_ direction because their q_⊥_-component intensity quickly dampens at high q_⊥_. Previous studies have demonstrated that intensity modulations along q_⊥_ are related to the correlation of heterogeneous density distributions along substrate normal direction [66,67]. In our study, the intensity modulations observed for the q_//_^par^ rods should be ascribed to the out-of-plane correlation of parallel cylinders along the substrate normal. In the P(S_21_-b-M_21_)/PS_6_ thin film, the perpendicular cylinders grew and propagated throughout the whole film thickness. Thus, the absence of intensity modulations for the q_//_^per^ rods suggests that the free surface and the substrate interface were uncorrelated in the perpendicular cylinders. For uncorrelated interfaces, it has been proposed that the roughness of a free surface is mainly determined by the surface topography of perpendicular nanodomains, while the roughness of a substrate interface is determined by an underlying substrate [66,67,68].

### 3.3. Surface Wrinkling for Perpendicular Lamellae

Previous studies [42,43,69] demonstrated that the surface property of a film can be altered by UV-light/ozone exposure. The skin surface of a film inevitably exhibits a different mechanical property from an inner region underneath the surface because the light dosage varies along the depth of the film. As a result, solvent swelling of such a film with two distinct mechanical properties frequently induces wrinkle patterns, whose period and amplitude are governed by the interplay between mechanical properties (such as moduli of a cross-linked skin layer and non-cross-linked inner region underlying it), swelling ratios by solvent, and thicknesses [42,43]. In other words, the periodicity (λ_c_) and amplitude of wrinkles strongly depend on properties of materials, given by [42,43].

(1) $$\lambda_{c}=2\pi h_{s}{\left[\frac{\left(1-v_{f}^{2}\right)E_{s}}{3\left(1-v_{s}^{2}\right)E_{f}}\right]}^{1/3}$$

where *h_s_* denotes the thickness of a skin layer; *E_s_*, *E_f_*, *v_s_* and *v_f_* are the elastic moduli and poisson’s ratios of the cross-linked skin layer and uncross-linked inner, respectively According to Equation (1), the wavelength of wrinkles can increase with increasing *h_s_*. In our study, *h_s_* could be increased by long duration of UV-light exposure.

**Figure 4:**
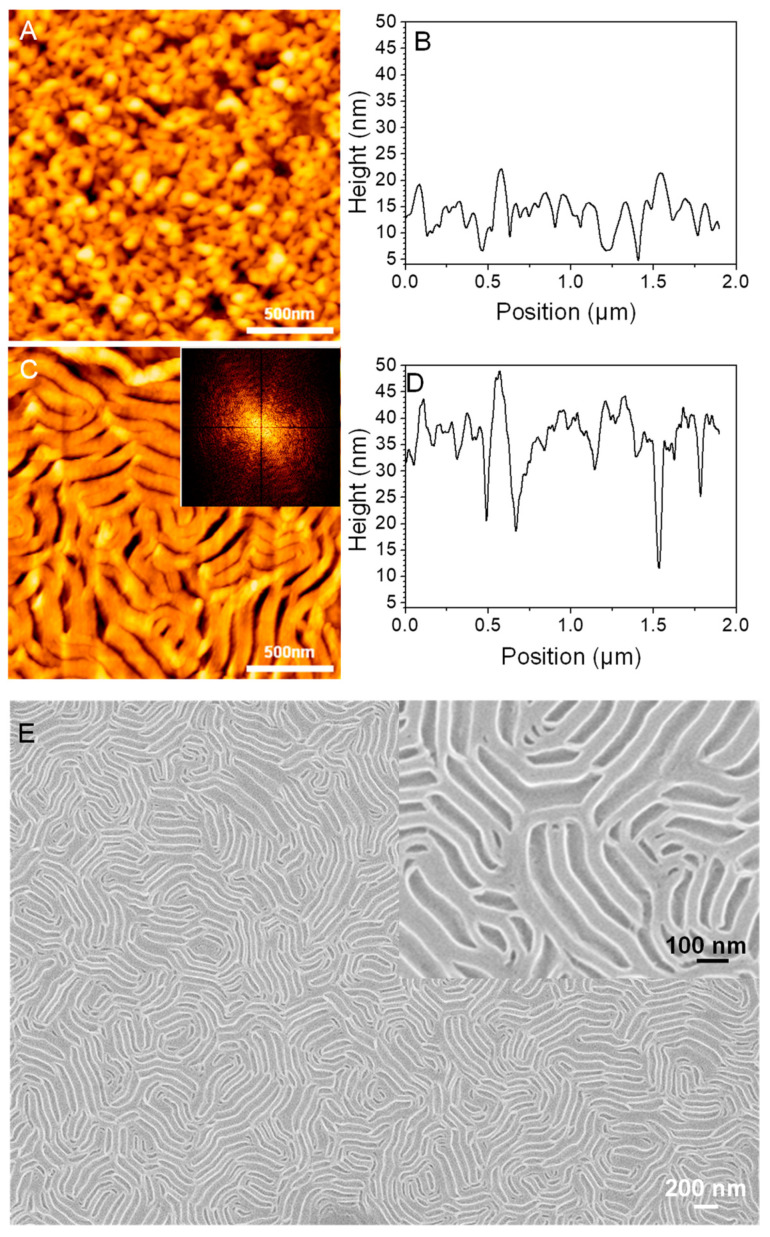
(**A**,**C**) AFM topographic images and (**B**,**D**) height profiles of neat P(S_21_-b-M_21_) films (h_i_ = 97 nm on PS_270_^n^-SiO_x_/Si) with perpendicular lamellae. Prior to AFM characterization, the films were briefly immersed in acetic acid (**A**,**B**) without and (**C**,**D**) with UV-light irradiation of 5 h. The inset in (**C**) denotes the corresponding FFT pattern of Image C. (**E**) low-magnification SEM image of the same film of Figure 4A,B. The insert in (**E**) is a large-magnification SEM image.

To study the effect of the duration of UV-light exposure on the periodicity wrinkles, we prepared two neat P(S_21_-b-M_21_) thin films that initially formed perpendicular lamellae on PS_270_^n^-SiO_x_/Si. One P(S_21_-b-M_21_) film was selected for UV-light exposure of 5 h followed by acetic acid rinsing. The other one was not exposed to UV-light but directly immersed in acetic acid for rinsing. As Figure 4A shows, most of the perpendicular lamellae without UV-light exposure collapsed after direct immersion in acetic acid. The collapse of perpendicular lamellae thus resulted in surface roughening with height variations being less than 10 nm (Figure 4B). In contrast, the perpendicular lamellae with prolonged UV-light exposure formed profound wrinkles (Figure 4C). The profound wrinkles have expanded widths and deep valleys (Figure 4D). This experiment verifies that a combination of UV-light exposure and acetic acid rinsing enabled surface wrinkling for perpendicular lamellae. Additionally, this result demonstrates that the periodicity and amplitude of the wrinkles could increase alongside increasing the duration of exposure. However, if the distance between two relief nano-stripes is extremely small due to expansion of PS nanodomains, the shallow nanogrooves resulted from PMMA removal may not be clearly probed with AFM (Figure 4C). Therefore, the relief nano-stripes seem to fuse together to form lamellar bundles with expanded width (see Figure 4C). Furthermore, the height and depth of the wrinkles fluctuate widely (Figure 4D). Thus, the wrinkles have polydisperse sizes. As a result, only a diffuse FFT pattern was present (see the inset of Figure 4C). Considering possible AFM tip-sample effects, we also carried out SEM to characterize the sample of Figure 4A,B. As the insert of Figure 4E shows, the perpendicular lamellae were tightly squeezed so that their packing is hardly probed, even under the SEM characterization. Instead, only the pattern of surface wrinkles with a large periodicity can be clearly observed in Figure 4E.

**Figure 5:**
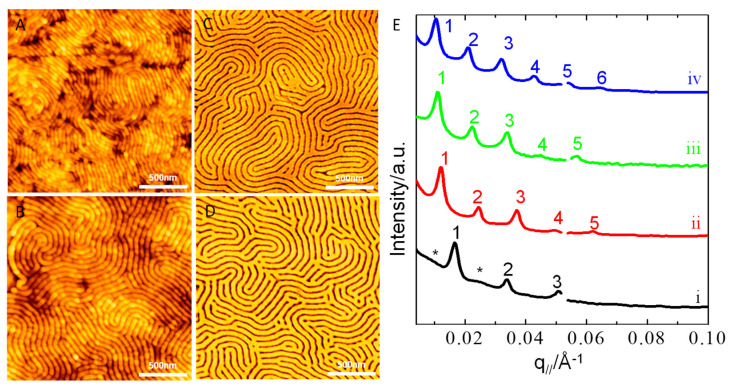
(**A**–**D**) 2 µm × 2 µm AFM topographies and (**E**) 1D in-plane GISAXS profiles of thin films (h_i_ = 97nm on PS_270_^n^-SiO_x_/Si) of neat P(S-b-MMA) BCPs of different molecular weights: (**A**,**E**_i_) P(S_33_-b-M_33_), (**B**,**E**_ii_) P(S_45_-b-M_44_), (**C**,**E**_iii_) P(S_53_-b-M_54_) and (**D**,**E**_iv_) P(S_65_-b-M_61_). The films were annealed at 230 °C (1 h), and treated by a combination of UV-light exposure (2.5 h) and acetic acid rinsing. In (**E**), lines i−iv correspond to the 1D GISAXS profiles extracted from in-plane scan cuts along the intense streaks. In curve−E_i_, broad humps corresponding to the scattering of wrinkles are marked by black asteroids (*). The positions of the diffraction truncation rods are labelled by q_//_ ratios.

Different molecular weight should have distinct mechanical properties. Therefore, in addition to the dosage of UV-light exposure, surface wrinkling should be influenced by BCP molecular weight. To study the effect of molecular weight in P(S-b-MMA) on surface wrinkling, we prepared thin films of neat P(S-b-MMA) BCPs of different molecular weights. After thermal annealing at 230 °C, the films formed perpendicular lamellae on PS_270_^n^-SiO_x_/Si. To induce osmotic pressure, the thermally annealed films were exposed to UV-light for 2.5 h followed by brief acetic acid rinsing. The films were characterized by AFM and GISAXS. As Figure 5 shows, the treatment of UV-light exposure and acetic acid rinsing only produced relief nano-stripes. The relief nano-stripes are due to PMMA removal. No wrinkling formed in films of P(S_45_-b-M_44_), P(S_53_-b-M_54_) and P(S_65_-b-M_61_) BCPs. In contrast, the film of P(S_33_-b-M_33_) could form wrinkles with small amplitude (Figure 5A). Due to small amplitude, the feature of surface wrinkling was not clearly probed by AFM but could be detected by GISAXS (Figure 5E_i_). In Figure 5E_i_, the broad humps were associated with surface wrinkling. This result indicates that using high-molecular-weight P(S-b-MMA) BCPs could prevent thin films from surface wrinkling during acetic acid rinsing.

**Figure 6:**
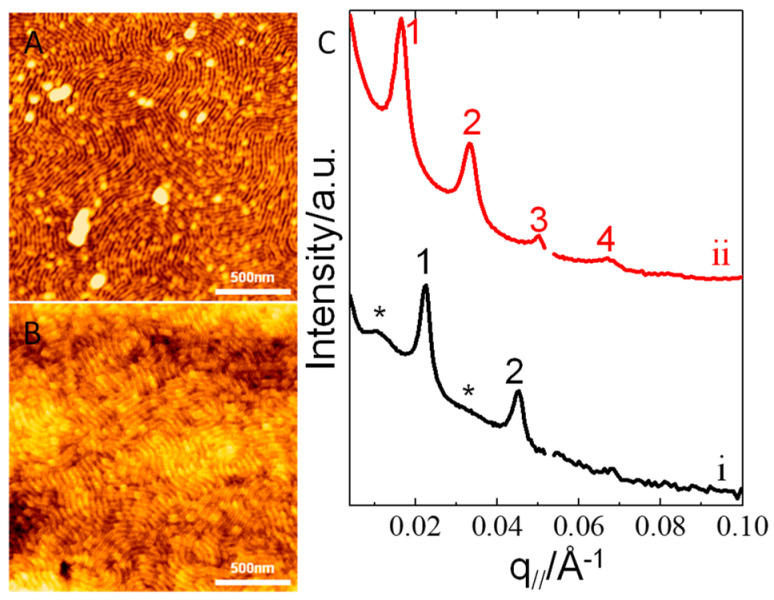
(**A**,**B**) 2 × 2 µm^2^ AFM topographies and (**C**) 1D in-plane GISAXS profiles of thin films (h_i_ = 17 nm on PS_270_^n^-SiO_x_/Si) of neat P(S-b-MMA) BCPs of various molecular weight: (**A**,**C_i_**) P(S_21_-b-M_21_) and (**B**,**C_ii_**) P(S_33_-b-M_33_). The films were annealed at 230 °C (1 h) and treated by a combination of UV-light exposure (2.5 h) and acetic acid rinsing. In curve−C_i_, broad humps corresponding to the scattering of wrinkles are marked by black asteroids (*). The positions of the diffraction truncation rods are labelled by q_//_ ratios.

To study the effects of film thickness on surface wrinkling, we also prepared another set of thin films (h_i_ = 16.3 nm) on PS_270_^n^-capped SiO_x_/Si for the neat P(S_21_-b-M_21_) and P(S_33_-b-M_33_) BCPs. After UV exposure and acetic acid rinsing, the surface morphology of the pristine film was characterized by AFM and GISAXS. As Figure 6A,B shows, the P(S_21_-b-M_21_) and P(S_33_-b-M_33_) films form nanogrooves in series as a result of PMMA removal. Additionally, the size of the nanogrooves is comparable to the dimension of the fingerprint-like nanodomains. This result suggests that osmotic pressure should not significantly expand the size of the perpendicular lamellae. Nevertheless, the corresponding GISAXS patterns of the nanogrooves for the P(S_21_-b-M_21_) film still displays a scattering of broad humps as a result of surface wrinkling (Figure 6C_i_). The scattering humps show weak intensity and coexist with Bragg truncation rods. The result indicates that the amplitude of the surface wrinkle in thin films is smaller than that in thick films. Furthermore, the broad scattering humps are located at q_//_ = 0.01107 Å^−1^. According to the position of the scattering lobes, the periodicity was estimated at approximately 56.8 nm. Both weak intensity and small periodicity suggest that although surface wrinkling could still form in thin films, the extent of surface wrinkling is limited by the thickness of the film.

On the basis of the morphological observations made above, we propose a mechanism of surface wrinkling triggered by osmotic pressure that occurs during immersion in acetic acid for the films with perpendicular lamellae. For surface wrinkling, two requirements must be satisfied: UV-light exposure and solvent swelling. The former requirement changes the mechanical and chemical properties of a film. The latter requirement imparts external force to trigger surface wrinkling.

**Figure 7:**
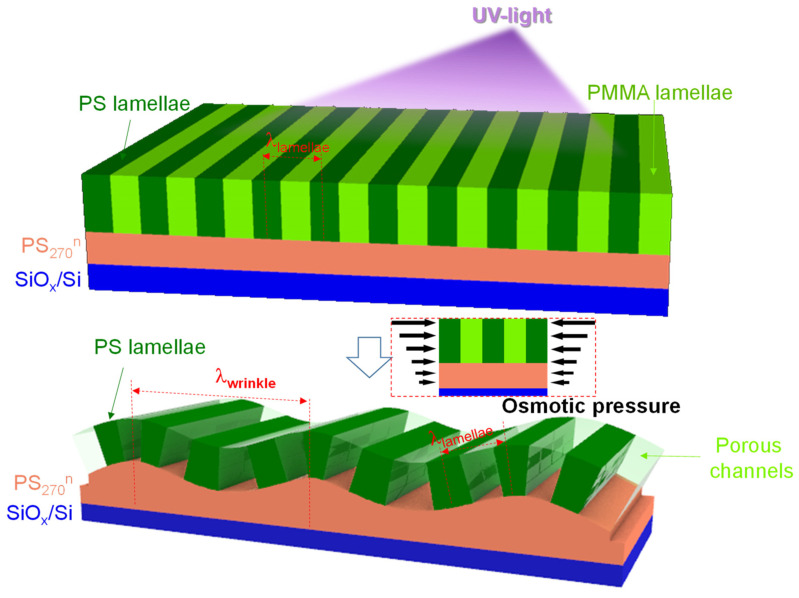
Schema of surface wrinkling of perpendicular lamellae in thin films of neat P(S-b-MMA) and its blends with a low content of PS during UV-light exposure followed by acetic acid rinsing. The parallel arrows shown in the middle diagrammatic sketch denote the magnitude gradient of osmotic pressure.

In this study, neat P(S-b-MMA), and its blend with a low content of PS, tend to form perpendicular lamellae on the neutralized substrate (see the top diagrammatic sketch of Figure 7). The main purpose of UV-light exposure is to remove the PMMA component through oxidative degradation. However, UV-light exposure also cross-links the PS component. Our previous study demonstrated that the cross-linking of the PS component initiates from chain-scission, which must produce free radicals with a concentration gradient [52,70]. The concentration gradient of free radicals is likely due to different distances to a UV-lamp and the attenuation of the UV-light by the top surface. The top surface and inner part of the films should experience disparate UV-light dosages. As a result, the free surface has the highest concentration of free radicals, whereas the bottom region in the vicinity of the substrate interface has the lowest concentration of free radicals. The free radicals are reactive species, which can react with either their analogous radicals or oxygen molecules. A recombination of free radicals leads to cross-linking and the reaction of free radicals with oxygen species results in oxidation. Similarly to the distribution of free radicals, the concentration of oxygen molecules is also gradient: highest on the free surface and lowest in the vicinity of the substrate interface. Thus, it is expected that the extents of oxidation and cross-linking depend on various depths within a film. Our result is correlates with previous studies [69,71], which have demonstrated that varied oxidation and cross-linking can lead to variations in property between the free surface and inner regions.

Next, we explain why acetic acid rinsing can result in surface wrinkling of the UV-light-exposed films that form perpendicular lamellae. The interpretation of the generation of osmotic pressure is based on the Tanaka et al.’s pioneering work [72]. When the thin films were immersed in acetic acid for rinsing, acetic acid could quickly swell the films. Solvent swelling always started from the outer surface and gradually diffused into the inner regions of the film. Thus, there should be a gradient in the concentration of acetic acid within the films. The bottom area, close to the rigid substrate, should remain confined and unchanged. By acetic acid rinsing, anisotropic osmotic pressure is caused by different concentrations of acidic acid along the thickness of the film (see the middle diagrammatic sketch of Figure 7). If the molecular weight of the PS-b-PMMA top layer is low, such anisotropic pressure can easily deform the outer surface to wrinkle, leading to patterns of surface wrinkles (see the bottom diagrammatic sketch of Figure 7). In fact, such anisotropic pressure can result in the formation of surface nanostructures [47], pores [48,49], and wrinkles [69,71] in thin films supported on a rigid substrate. We also notice that surface instability might also be induced by capillary forces that may emerge during drying [73]. However, the mechanism of drying-induced capillary forces might not cause periodic wrinkles in films with perpendicular lamellae.

## 4. Conclusions

We have demonstrated the phase behavior of thin films with mixtures of a symmetric weakly-segregated P(S_21_-b-M_21_) and PS homopolymer, at various weight fractions. Upon blending PS at different weight fractions, perforated lamellae and cylinders were obtained in thin films of the P(S_21_-b-M_21_)/PS mixtures. A combination of UV-light exposure and acetic acid rinsing was used to remove the PMMA block. Interestingly, the post-treatment produced surface wrinkling for perpendicular lamellae. Such surface wrinkling is due to the interplay between UV-light exposure and acetic acid rinsing. As a result, wrinkled films with perpendicular lamellae formed a hierarchical structure with two periodicities. Nevertheless, the surface wrinkling could be suppressed by reducing the film thickness, increasing the PS content and using high P(S-b-MMA).

## Figures and Tables

**Table 1:** Details of P(S-*b*-MMA) BCPs and PS Homopolymers.

Materials	M_n_^total^ (kg/mol)	M_n_^PS^ (kg/mol)	M_n_^PMMA^ (kg/mol)	M_w_/M_n_	*f*_PS_	Morphology
P(S_65_-b-M_61_)	126	65	61	1.10	0.51	lamellae
P(S_53_-b-M_54_)	107	53	54	1.16	0.49	lamellae
P(S_45_-b-M_44_)	89	45	44	1.12	0.49	lamellae
P(S_33_-b-M_33_)	66	33	33	1.09	0.5	lamellae
P(S_21_-b-M_21_)	42	21	21	1.07	0.5	lamellae
PS_270_	270			1.06	1	
PS_6_	6.1			1.33	1	

## Data Availability

The data presented in this study are available on request from the corresponding author.

## Supplementary Materials

The following are available online at https://www.mdpi.com/article/10.3390/polym13152480/s1. Figure S1.FFT patterns of Figure 1. Figure S2. Height profiles of Figure 1.
