# Supplementary material for: Influence of Osmotic Pressure on Nanostructures in Thin Films of a Weakly-Segregated Block Copolymer and Its Blends with a Homopolymer
Source: Polymers (Basel). 2021 Jul 28;13(15):2480. doi: 10.3390/polym13152480 (PMC8348333; doi:10.3390/polym13152480)
Supplement: Supplementary file 1 [file polymers-13-02480-s001.zip › polymers-1291681-supplementary.pdf]

Supporting information

Influence of Osmotic Pressure on Nanostructures in Thin Films of a Weakly-Segregated Block Copolymer and its Blends with a Homopolymer.

*Yi-Fang Chen, Jia-Wen Hong, Jung-Hong Chang, Belda Amelia Junisu, Ya-Sen Sun**

Department of Chemical and Materials Engineering, National Central University, Taoyuan 32001, Taiwan

*corresponding author: Y. S. Sun (Email: yssun@cc.ncu.edu.tw)

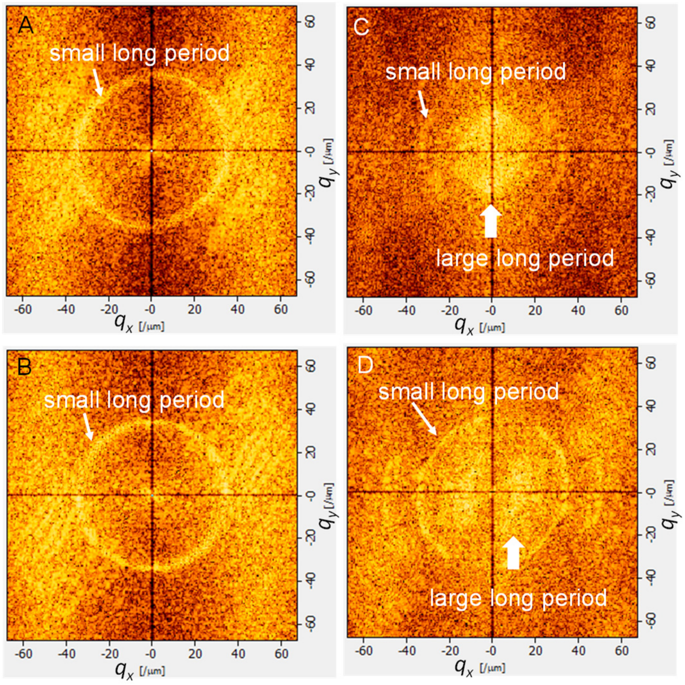

Figure S1. (A, B) Fast Fourier transform (FFT) patterns of Figure 1A and 1B, and (C, D) FFT patterns of Figure 1E and 1F.

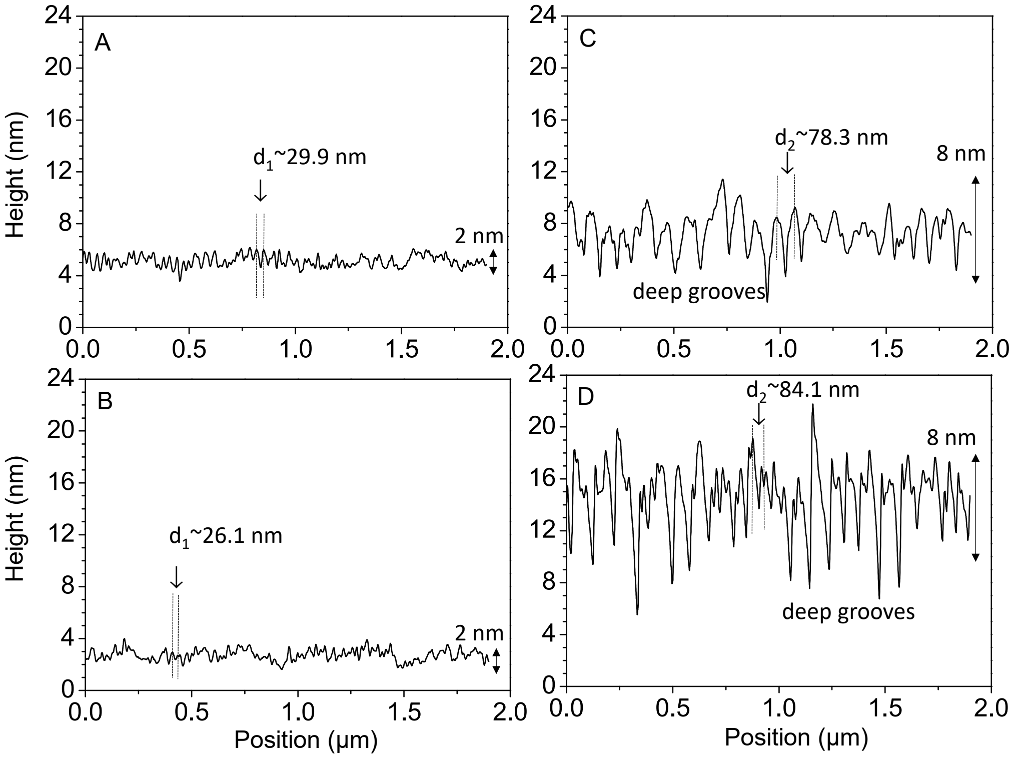

Figure S2. (A, B) Height profiles of Figure 1A and 1B, and (C, D) height profiles of Figure 1E and 1F.
